# Protocol to study toxic, immune, and suicidal phenotypes of killer yeast

**DOI:** 10.1016/j.xpro.2025.103611

**Published:** 2025-01-31

**Authors:** Rianne C. Prins, Sonja Billerbeck

**Affiliations:** 1 Molecular Microbiology, Groningen Biomolecular Sciences and Biotechnology Institute, University of Groningen, Groningen 9747 AG, the Netherlands; 2 Department of Bioengineering, Imperial College London, London SW7 2AZ, UK

**Keywords:** microbiology, molecular biology, biotechnology and bioengineering

## Abstract

Killer yeast produce antimicrobial proteins, often together with a self-protective immunity factor. Here, we present a protocol for analyzing the toxic, immune, and suicidal phenotypes in *Saccharomyces cerevisiae*. We describe steps for assessing toxicity via halo assays, suicidality through spot assays, and immunity using microtiter plate growth assays. We detail procedures for yeast culturing, performing the assays, and data analysis.

For complete details on the use and execution of this protocol, please refer to Prins et al.^1^

## Highlights

• Workflow to determine toxic, immune, and suicidal phenotypes of yeast
• Assess potential toxin secretion by a simple and robust halo assay
• Determine various immune phenotypes by exposing yeast strains to externally added toxin
• Identify suicidal phenotypes in spot assays

## Abstract (editor highlights)

Publisher’s note: Undertaking any experimental protocol requires adherence to local institutional guidelines for laboratory safety and ethics.

## Graphical abstract

**Figure:**
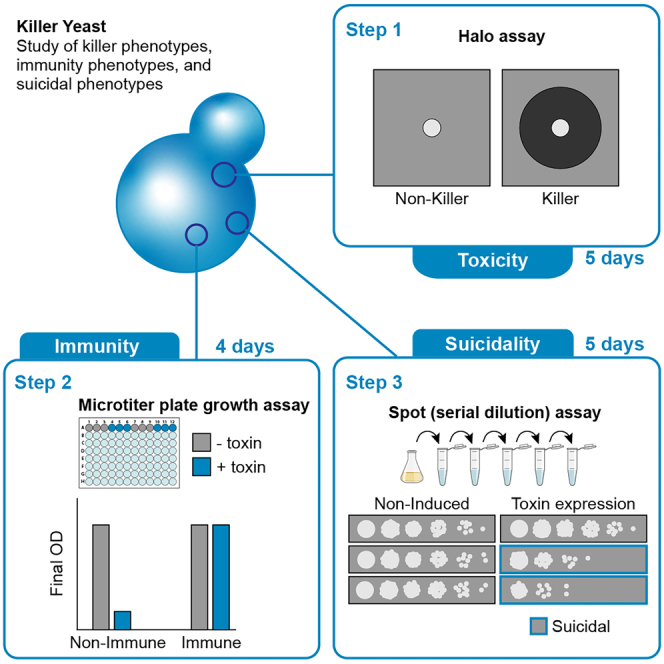

## Before you begin

The following protocol describes assays for studying killer yeast phenotypes. The protocol has been used to investigate active toxin production and self-immunity related to the K2 killer toxin in *Saccharomyces cerevisiae.*^1^ In addition, we have used parts of this protocol to study secretion of and resistance to other toxic compounds in yeast, as well as in other yeast species,^2^ including *Nakaseomyces glabratus*.

Because killer yeast often produce a toxin together with a self-protective immunity factor, three assays need to be performed in tandem to provide a complete picture of the toxic, immune or potential suicidal phenotypes that are associated with the production of a toxin or an (engineered) toxin variant. The first part of the protocol describes steps for studying functional toxin secretion using a halo assay, which is based on the formation of a zone of inhibition on a lawn of a sensitive indicator strain. The second part of the protocol describes how immune phenotypes can be determined in cells that produce an immunity factor, using growth assays in microtiter plates in the presence or absence of an externally added toxin. The third part of the protocol includes the steps for performing a spot assay to assess a potential suicidal phenotype, which can result from toxin production but lack of a cognate self-protection factor.

As the study of yeast killer toxins often requires specific pH and temperature ranges for a toxin to be active, we will use buffered media^3^ and controlled incubation temperatures (25°C, 30°C) throughout this protocol. Note that such parameters may vary depending on the specific toxin under investigation. Further, we use a galactose-inducible promoter to enable switching protein expression off or on.

Before starting, order all required reagents and consumables for yeast culturing (see: key resources table). In addition, prepare all necessary media components (see: materials and equipment setup). All assays described in this protocol can be performed using standard laboratory equipment. The assays can be performed in parallel within one week.

***Note:*** Access to a microtiter plate reader with shaking and temperature control is recommended for facilitating high-throughput generation of time-resolved growth data for the immunity assay, but the assay can also be performed in individual culture tubes.

***Note:*** For the spot (serial dilution) assay, it is critical that toxin expression is inducible. Alternatively, physicochemical parameters may be used to repress toxin activity such as pH or temperature.

### Preparing yeast patches

**Timing: 0.5 h + 2–3 days for growth**

Start by creating yeast patches to yield sufficient biomass of the strains of interest. By creating yeast patches from single colonies, we generate sufficient starting material to perform multiple experiments.

**Figure 1:**
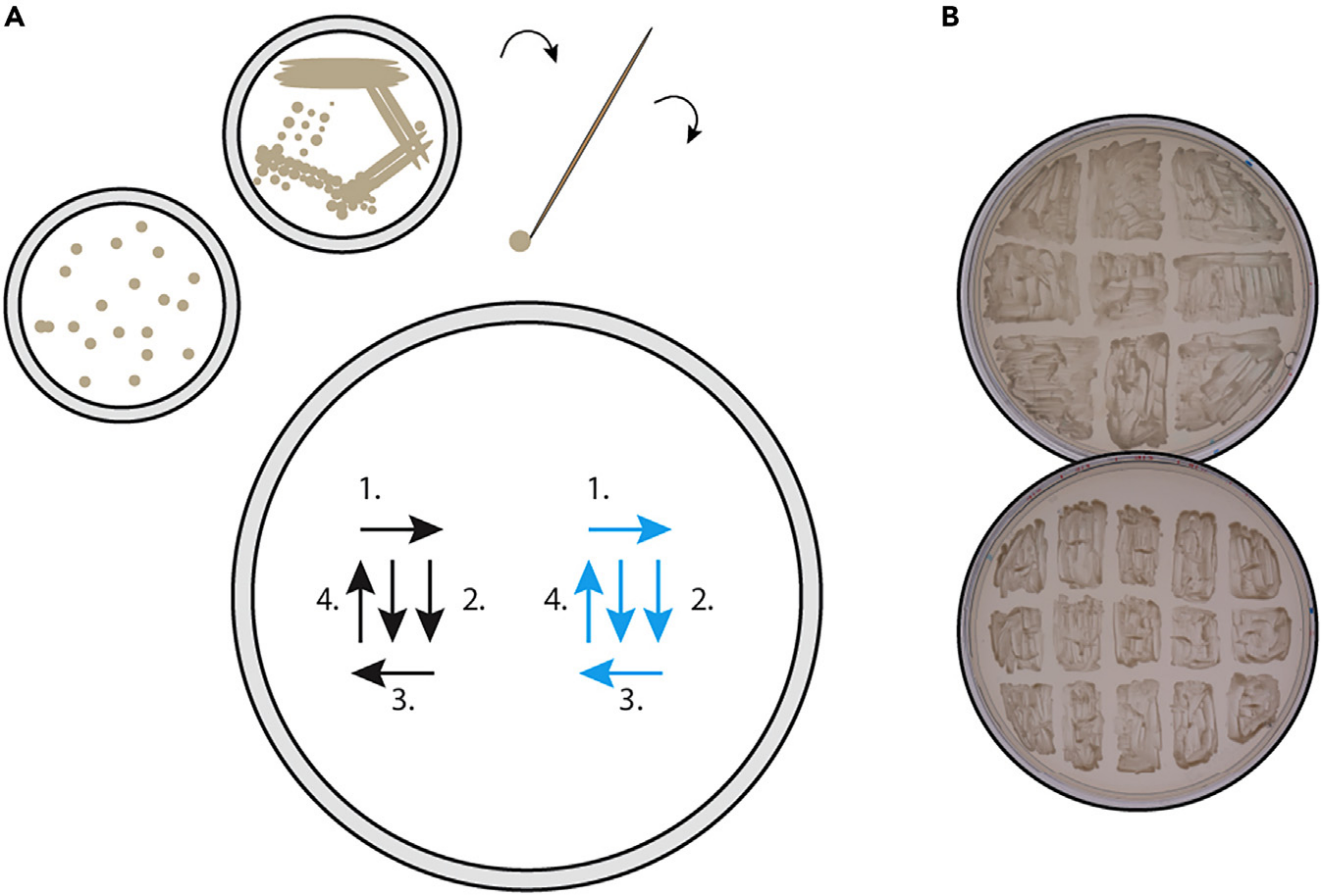
Creating patches from single colonies (A) Streak single colonies or transformants on a plate in a square using sterile toothpicks. Repeat this process for each strain of interest. Incubate the plate for 2–3 days until the patch has grown. (B) Examples of plates with patched yeast strains in triplicates. Transformants of the yeast *S. cerevisiae* were grown on selective SD_gluc_ agar plates at 30°C for 3 days.

1. Streak the yeast strains of interest (e.g., those potentially secreting an active toxin and those used as indicator strains) onto appropriate media plates.
  a. With a sterile toothpick, take a single colony (e.g., from a streaked plate or a transformation plate, Figure 1A) and transfer it to a fresh plate.
  b. With the same toothpick, spread the cells into a square (approximately 1–2 cm) on the fresh plate to create a ‘patch’.
  c. Repeat this for each strain of interest, creating the desired number of replicate patches for each strain.
  d. Incubate the plate at 30°C for 2–3 days. See example in Figure 1.

***Note:*** Incubation temperature and time are not critical for this step and can be adjusted depending on the growth needs of the used strains.

**Pause point:** Multiple patches can be combined on a single plate and these plates can be stored at 4°C for several weeks until use.

### Preparing concentrated killer toxin for the immunity assay

**Timing: 4 days**

For the immunity assay, we prepare a concentrated killer toxin sample from the spent culture media of a toxin producing yeast. By concentrating the proteins in this spent culture media using centrifugal filter units with a molecular weight cutoff, a batch of the killer toxin concentrate is obtained.

**Figure 2:**
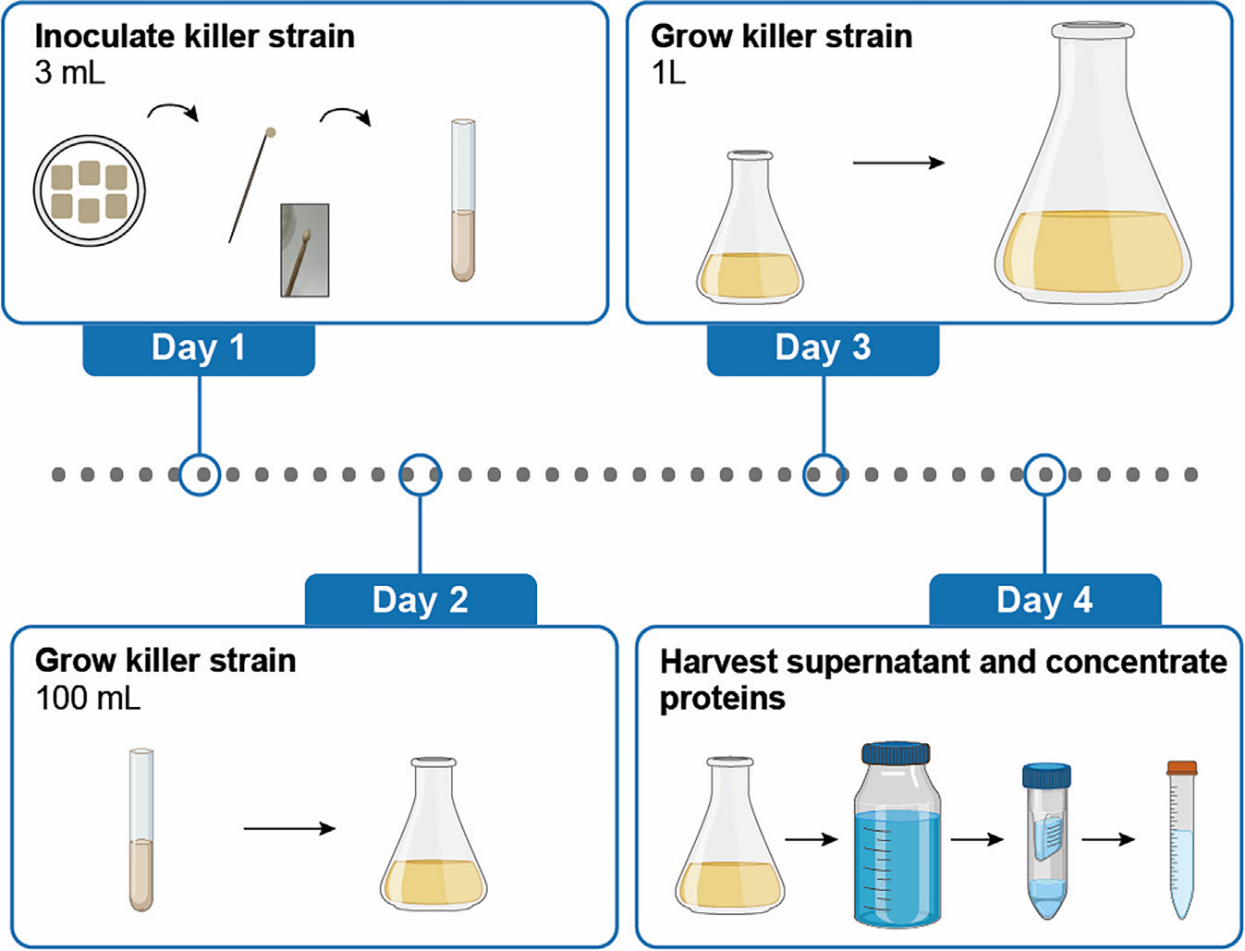
Preparing a batch of yeast killer toxin concentrate Day 1: Inoculate 3 mL of media by scraping the killer yeast from the patch and transferring it into the culture tube. Day 2: After overnight incubation, inoculate a larger volume to create more biomass. Day 3: Repeat to reach the desired culture volume of 1 L. Day 4: Cool the culture on ice. Harvest the spent culture media by pelleting the cells, sterile filtering the spent culture media, and concentrating the proteins in the spent culture media using filter units with an appropriate size cutoff (in kDa).

2. Grow an appropriate volume of the killer toxin producing yeast in a shake flask (Figure 2).
  ***Note:*** We use a strain containing a plasmid for constitutive toxin production and secretion, and grow the strain in SD_suc_ media (without galactose or glucose).
  ***Note:*** We assume it has been determined by a user under which growth conditions and at what optical density (OD_600_) the killer yeast yields the highest toxin concentration in the spent culture media (e.g. as determined by halo assay). For example, in our case we grow the strain to OD_600_ 6.0 (approximately 1.2∗10^8^ cells per mL).
  a. Day 1: Scrape an appropriate amount of biomass from the yeast patch of the killer toxin producer and add this to 3 mL of SD_suc_ media in a glass culture tube to grow the strain overnight (∼16 h) at 25°C, 200 rpm.
  b. Day 2: Transfer the 3 mL culture to a new shake flask with 100 mL SD_suc_ media, and grow overnight at 25°C, 200 rpm.
  c. Day 3: Inoculate a shake flask containing 1 L of SD_suc_ media using the overnight culture. The starting OD_600_ should be chosen, such that the optimal OD_600_ for harvesting is reached after overnight growth. Grow overnight at 25°C, 200 rpm. For example, in our case we inoculated at OD_600_ 0.25 and harvested after ∼17 h of growth.
3. Day 4: Collect the spent culture media – containing the secreted toxin - and sterile-filter it.
  a. Cool the culture on ice and keep at 4°C during the following steps.
  b. Pellet the cells by centrifugation (e.g., 5000 *g* for 10 min).
  c. Sterile filter the spent culture media into a sterile glass bottle using a bottle-top filter unit.
    **Pause point:** This sterile filtered spent culture media containing the toxin can be stored at 4°C without losing killer toxin activity. This is true for the K2 toxin in the above indicated SD_suc_ media, but would need to be verified for new killer toxins and/or other media types.
  d. Using Amicon centrifugal filter units with an appropriate size cutoff (we use 10 kDa for the K2 toxin), concentrate the spent culture media to an appropriate fold concentration (e.g., 100X) according to manufacturers’ instructions.
  e. Collect the concentrated toxin from the centrifugal unit and transfer to a sterile 15 mL falcon tube. For the K2 toxin, we determined that this toxin concentrate stays active when stored at 4°C for several months.
    ***Note:*** For yeast killer toxins, there may not always be accurate quantification methods available to determine the toxin concentration. Although this protocol in our hands results in reproducible phenotypes, to prevent some batch-to-batch toxin concentration variation to influence experiments, we recommend to use toxin concentrate from a single batch per experiment. If multiple toxin concentrate batches need to be used to yield sufficient material for an experiment, we recommend first combining the batches together into a single batch, and then applying it to the samples within the experiment, such that it is guaranteed that the toxin concentration will be equal across all samples.

## Key resources table

**Table:** 

REAGENT or RESOURCE	SOURCE	IDENTIFIER
**Chemicals, peptides, and recombinant proteins**		

ddH_2_O	N/A	N/A
Glucose	Sigma-Aldrich	1.08342
Sucrose	Carl Roth	4621.1
Galactose	Thermo Scientific	150615000
Agar	Becton Dickinson	214530
Adenine sulfate	Sigma-Aldrich	A8626
Arginine HCl	Sigma-Aldrich	A5131
Aspartic acid	Sigma-Aldrich	1.00126
Glutamic acid	Sigma-Aldrich	G1251
Isoleucine	Carl Roth	1698.2
Lysine HCl	Sigma-Aldrich	L5626
Methionine	Carl Roth	1702.2
Phenylalanine	Sigma-Aldrich	P2126
Serine	Carl Roth	1714.2
Threonine	Carl Roth	1738.2
Tyrosine	Carl Roth	1741.2
Valine	Carl Roth	1742.2
Tryptophan	Sigma-Aldrich	1.08374
Histidine	Sigma-Aldrich	53370
Leucine	Sigma-Aldrich	L8000
Uracil	Acros Organics	157301000
Yeast nitrogen base	Sigma-Aldrich	Y1251
Yeast nitrogen base w/o ammonium sulfate	Thermo Scientific	H26271
Urea	Sigma-Aldrich	1.08487
Na_2_HPO_4_	Sigma-Aldrich	1.06580
Citric acid	Sigma-Aldrich	1.00244

**Experimental models: Organisms/strains**		

*Saccharomyces cerevisiae* BY4741	ATCC	201388

**Recombinant DNA**		

pRP001 (constitutive K2 toxin producer)	Prins et al., 2024^1^	N/A
pRP223 (wild-type K2 [WT K2])	Prins et al., 2024^1^	N/A
pRP246 (empty plasmid [EP])	Prins et al., 2024^1^	N/A
pRP248 (variant 1)	Prins et al., 2024^1^	N/A
pRP249 (variant 2)	Prins et al., 2024^1^	N/A
pRP250 (variant 3)	Prins et al., 2024^1^	N/A
pRP251 (variant 4)	Prins et al., 2024^1^	N/A
pRP252 (variant 5)	Prins et al., 2024^1^	N/A
pRP232 (variant 6)	Prins et al., 2024^1^	N/A
pRP075 (variant 7)	Prins et al., 2024^1^	N/A

**Software and algorithms**		

ImageJ	Schneider et al.^4^	https://imagej.net/

**Other**		

Nalgene Rapid-Flow bottle-top filter units (0.2 μm)	Thermo Scientific	566-0020
Amicon centrifugal filter units (10 kDa)	Sigma-Aldrich	UFC5010
Petri dishes	different vendors	N/A
Greiner OneWell plates	Sigma-Aldrich	670102
Falcon tubes 50 mL	Sarstedt	62.547.254
Falcon tubes 15 mL	Sarstedt	62.554.502
Greiner CELLSTAR transparent round-bottom 96-well microtiter plates	Sigma-Aldrich	650180
Transparent Breathe-Easy membranes	Diversified Biotech	BEM-1
Autoclaved toothpicks	N/A	N/A
Plate reader	BioTek Synergy MX	N/A
Multichannel pipette (optional)	Gilson	N/A

## Materials and equipment

**Table:** 500 mL of 20X amino acids (AA) stock solution (sterile filtered)

Reagent	Final concentration (in 20X)	Amount (g)
Adenine Sulfate	400 mg/L	0.2
Arginine HCL	400 mg/L	0.2
Aspartic acid∗	2 g/L	1
Glutamic acid	2 g/L	1
Isoleucine	600 mg/L	0.3
Lysine HCl	600 mg/L	0.3
Methionine	400 mg/L	0.2
Phenylalanine	1 g/L	0.5
Serine	8 g/L	4
Threonine∗	4 g/L	2
Tyrosine	600 mg/L	0.3
Valine	3 g/L	1.5
*Total*	*23 g/L*	*11.5*

Store at room temperature (RT, range: 20°C–25°C) for up to 6 months

∗ heat sensitive – add up to 500 mL ddH_2_O and stir the solution under light heating until it is fully dissolved, then sterile filter it using a bottle-top filter unit with a 0.2 μm pore size.

### Sterile ddH_2_O (autoclaved)

• Add 500 mL deionized water into a bottle.
• Autoclave (20 min, 121°C).

Store at RT for up to 6 months.

### 8X SD (autoclaved)

• Add 26.8 g Yeast Nitrogen Base without amino acids with ammonium sulfate to 500 mL ddH_2_O.
• Autoclave.

Store at RT for up to 6 months.

### 10X SD (autoclaved)

• Add 8.5 g Yeast Nitrogen Base without amino acids *and without* ammonium sulfate to 500 mL ddH_2_O.
• Autoclave.

Store at RT for up to 6 months.

### 40% glucose (sterile filtered)

• 40% (w/v): Add 40 g glucose in a bottle, fill up to 100 mL with ddH_2_O.
• To aid dissolving, heat up the water first, and dissolve while stirring.
• Once dissolved, sterile filter (0.2 μm pore size) into a sterile bottle.

Store at RT for up to 6 months.

### 40% galactose (sterile filtered)

• 40% (w/v): Add 40 g galactose in a bottle, fill up to 100 mL with ddH_2_O.
• Heat up the water first, add to the galactose powder and dissolve while stirring.
• Sterile filter into a sterile bottle (0.2 μm pore size).

Store at RT for up to 6 months.

### 30% sucrose (sterile filtered)

• 30% (w/v): Add 30 g sucrose to a bottle, fill up to 100 mL with ddH_2_O.
• Heat up the water first, add to the sucrose powder and dissolve while stirring.
• Sterile filter into a sterile bottle (0.2 μm pore size).

Store at RT for up to 6 months.

### 0.2% uracil (autoclaved)

• Add 0.4 g to 200 mL ddH_2_O.
• Autoclave.

Store at RT for up to 6 months.

### 1% histidine (autoclaved)

• Add 2 g to 200 mL ddH_2_O.
• Autoclave.

Store at RT for up to 6 months.

### 1% tryptophan (autoclaved)

• Add 2 g to 200 mL ddH_2_O.
• Autoclave.

***Note:*** Protect from light by wrapping in aluminum foil after autoclaving.

Store at RT for up to 6 months.

### 1% leucine (autoclaved)

• Add 2 g to 200 mL ddH_2_O.
• Autoclave.

Store at RT for up to 6 months.

### 4% agar (autoclaved)

• 4% (w/v): add 12 g agar in a 500 mL bottle, fill up to 300 mL with ddH_2_O.
• Autoclave.

Store at RT for up to 6 months.

***Note:*** After autoclaving, this solution will cool down and solidify. Either use it directly or heat and melt the 4% agar in a microwave before use.

### 1 M Na_2_HPO_4_ (autoclaved)

• 141.96 g/mol: Add 70.98 g into a 500 mL bottle, fill up to 500 mL with ddH_2_O
• Autoclave

Store at RT for up to 6 months.

### 1 M citric acid (autoclaved)

• 192.12 g/mol: Add 96.06 g into a 500 mL bottle, fill up to 500 mL with ddH_2_O.
• Autoclave.

Store at RT for up to 6 months.

### 10X urea (autoclaved)

• Add 25 g into a 500 mL bottle, fill up to 500 mL with ddH_2_O.
• Autoclave.

Store at RT for up to 6 months.

**Table:** Buffer ratios per 100 mL of 1x media (0.5x final buffer concentration^3^)

pH	1 M Na_2_HPO_4_ (mL)	1 M citric acid (mL)	Total volume (mL)
**2.2**	0.200	4.900	5.100
**2.4**	0.620	4.690	5.310
**2.6**	1.090	4.455	5.545
**2.8**	1.585	4.208	5.793
**3.0**	2.055	3.973	6.028
**3.2**	2.470	3.765	6.235
**3.4**	2.850	3.575	6.425
**3.6**	3.220	3.390	6.610
**3.8**	3.550	3.225	6.775
**4.0**	3.855	3.073	6.928
**4.2**	4.140	2.930	7.070
**4.4**	4.410	2.795	7.205
**4.6**	4.675	2.663	7.338
**4.8**	4.930	2.535	7.465
**5.0**	5.150	2.425	7.575
**5.2**	5.360	2.320	7.680
**5.4**	5.575	2.213	7.788
**5.6**	5.800	2.100	7.900
**5.8**	6.045	1.978	8.023
**6.0**	6.315	1.843	8.158
**6.2**	6.610	1.695	8.305
**6.4**	6.925	1.538	8.463
**6.6**	7.275	1.363	8.638
**6.8**	7.725	1.138	8.863
**7.0**	8.235	0.883	9.118
**7.2**	8.695	0.653	9.348
**7.4**	9.085	0.458	9.543
**7.6**	9.365	0.318	9.683
**7.8**	9.575	0.213	9.788
**8.0**	9.725	0.138	9.863

**Table:** For 500 mL of a 2X glucose media stock solution (2X SD_gluc_)

Reagent	Final concentration (in 2X)	Amount (mL)
10x SD without ammonium sulfate	1.7 g/L	50.0
10x Urea	5 g/L	50.0
8x SD	7.13 g/L	66.5
40% glucose	4%	50.0
20x amino acids	2.3 g/L	50.0
*1% Histidine*	40 mg/L	2.0
*1% Tryptophan*	40 mg/L	2.0
*0.2% Uracil*	40 mg/L	10.0
*1% Leucine*	180 mg/L	9.0
Sterile ddH_2_O	N/A	210.5
**Total**	N/A	**500 mL**

Store at RT for up to 6 months.

***Note:*** For selective media based on auxotrophy, leave out histidine, tryptophan, uracil and/or leucine if applicable and replace with additional ddH_2_O.

***Note:*** 2X means that all ingredients have been added in double amounts when compared to the regular media (1X). From a 2X concentrated media, both 1X liquid media or 1X solid media can be prepared.

**Table:** For 500 mL of a 2X buffered sucrose media stock solution (2X SD_suc_)

Reagent	Final concentration (in 2X)	Amount (mL)
10x SD without ammonium sulfate	1.7 g/L	50.0
10x Urea	5 g/L	50.0
8x SD	7.13 g/L	66.5
30% sucrose	4%	66.7
20x amino acids	2.3 g/L	50.0
*1% Histidine*	40 mg/L	2.0
*1% Tryptophan*	40 mg/L	2.0
*0.2% Uracil*	40 mg/L	10.0
*1% Leucine*	180 mg/L	9.0
1 M Na_2_HPO_4_	pH dependent	pH dependent
1 M citric acid	pH dependent	pH dependent
Sterile ddH_2_O	N/A	Up to 500
**Total**	N/A	**500 mL**

Store at RT for up to 6 months.

***Note:*** For selective media based on auxotrophy, leave out histidine, tryptophan, uracil and/or leucine if applicable and replace with additional ddH_2_O.

***Note:*** Since pH is critical for killer toxin activity, it is good practice for buffered media to confirm the correct pH of the stock media solution using pH indicator strips or an instrument for pH measurement.

**Table:** Ratios for mixing liquid culturing media

Reagent	Final concentration	Amount (mL)
2X media	1X	250
Sterile ddH_2_O	N/A	250
**Total**	N/A	**500 mL**

Store at RT for up to 6 months.

***Note:*** If additional components are added, adjust the ddH_2_O volume that is added.

**Table:** Ratios for mixing solid media

Reagent	Final concentration	Amount (mL)
2X media	1X	250
4% agar	1.6%	200
Sterile ddH_2_O	N/A	50
**Total**	N/A	**500 mL**

Plates can be stored at 4°C for up to 3 months.

***Alternatives:*** The chemicals listed in the key resources table can be replaced with identical chemicals of the same grade from different suppliers.

## Step-by-step method details

### Part 1: Assessing secretion of active yeast killer toxin by a halo assay

**Timing: 2 days + 2–3 days incubation**

**Figure 3:**
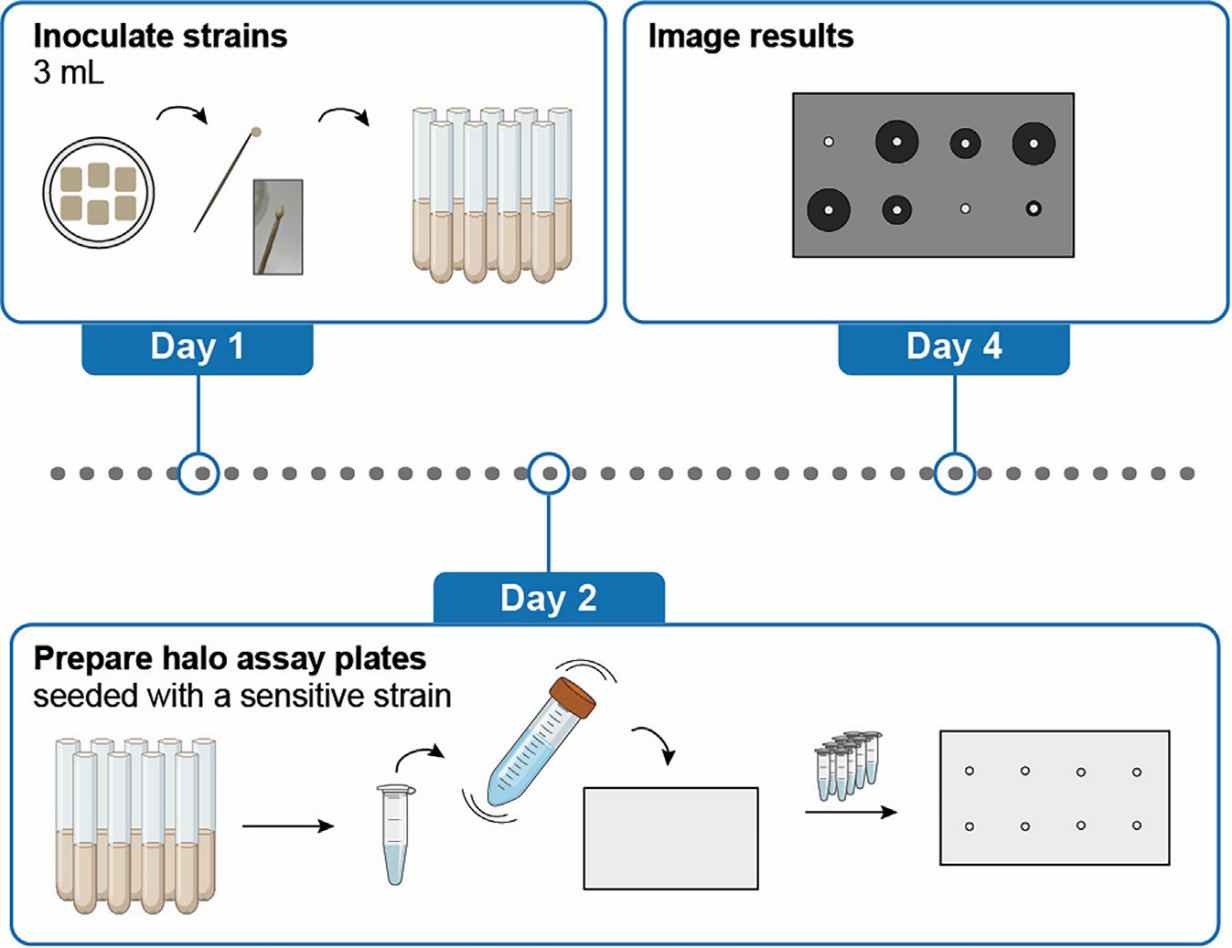
Process of performing a halo assay Day 1: Inoculate the strains of interest to create source cultures for the assay. Day 2: Adjust the OD_600_ of the cultures to 10. Add the indicator strain to the liquid media components in a falcon tube, add warm 4% agar, nutate, and pour into a petri-dish. Spot the potential killer strains on top of the agar. Day 4: Zones of inhibition are visible and can be further analyzed.

This section of the protocol describes how one can analyze whether a certain yeast strain secretes an active toxin (Figure 3). Additionally, this assay can be used to determine the level of sensitivity or resistance of an embedded indicator strain to the killer strain. Here, we describe an assay where toxin production is regulated by a galactose-inducible promoter.

***Note:*** In the halo assay, potential killer yeast strains are spotted onto an agar plate that contains a sensitive indicator strain embedded within the agar. If the killer strain secretes an active toxin, a transparent zone of inhibition (halo) will form around the spot, indicating where the sensitive strain cannot grow.

***Note:*** When studying variants of killer toxins, the size of the inhibition zone is related to the remaining functionality of the toxin. This functionality encompasses many aspects, from translation, to production, to processing, to secretion and diffusion, stability, and toxin interaction with a target cell – any impacted aspect can influence the size of the zone of inhibition.

1. Day 1: Grow the strains of interest.
  a. Prepare 1x buffered SD_suc_ medium supplemented with the inducer galactose (1% final concentration) (for media recipe see: materials and equipment setup).
  b. Prepare culture tubes containing 3 mL of this medium.
  c. Inoculate the strains of interest, including a positive and negative control and a sensitive indicator strain.
    i. Prepare duplicate or triplicate cultures for each strain.
    ii. Use sterile toothpicks to take some biomass from the respective patches to inoculate.
    iii. Incubate the cultures at 25°C, 200 rpm, overnight.

***Note:*** In our experience growing the strains of interest overnight in inducing media yields increased zones of inhibition when compared to overnight growth in non-inducing media.

2. Day 2: Normalize the OD_600_ of all cultures.
  a. Determine the OD_600_ of all cultures using a spectrophotometer.
  b. Adjust the cell concentration of all overnight cultures to an OD_600_ of 10.
    i. Transfer 1 mL of the cultures into Eppendorf tubes.
    ii. Pellet the cells by centrifugation (i.e., 5000 *g* for 2 min).
    iii. Adjust the OD_600_ by removing an appropriate volume of the spent culture media or adding an appropriate volume if the OD_600_ is above 10.
    iv. Vortex the tubes to resuspend the cell pellet.

***Note:*** For consistent results, it is important that the overnight OD_600_ values of the strains are similar (i.e. in the same growth phase). If there are significant differences, consider repeating by starting again at step 1.

***Note:*** For the calculation to adjust OD_600_ to 10 one can use the following formula: Desired OD_600_/measured OD_600_ = concentration factor (cf). 1 mL/cf = volume in which the cell pellet has to be resuspended. For example: if the OD_600_ of the overnight culture is 6, the cf is 1.67, and 1 mL/1.67 = 0.6 mL. Meaning you resuspend the pellet in 600 μL - from the 1 mL culture, 400 μL spent culture media is removed to reach an OD_600_ of 10.

3. Day 2: Prepare solid media assay plates seeded with a sensitive indicator strain.
  a. Heat the 4% agar solution and let it cool down on the bench before use.
  b. For filling one one-well rectangular plate (which fits 30 mL of media), take a 50 mL falcon tube and add all components to the falcon tube except for the indicator strain and the 4% agar (see Table below).
    ***Note:*** The number of required plates depends on the number of strains that will be tested and on the expected sizes of the zones of inhibition (for the K2 toxin producer with halo diameters of ∼1.2 cm we generally place 15 spots per one-well plate).
    **CRITICAL:** Make sure that the 4% agar solution is not too hot, or it will kill the cells, but also not too cold, or the media will quickly solidify and form clumps. As a rule of thumb, the right temperature is reached when you can comfortably hold the 4% agar bottle in your hands.
  c. Add the indicated volume of the indicator strain into the falcon tube.
  d. Pour the 4% agar into the falcon tube, close the tube, and gently but swiftly nutate a few times to mix the solution. Pour the solution into the one-well plate, ensuring an even distribution across the plate.
  e. Leave the plate on the bench until it is solid.

**Table:** Amounts for preparing solid media seeded with a sensitive indicator strain

Reagent	Final concentration	Amount (mL)
2X buffered SD_suc_	1X	15
40% galactose	1%	0.75
4% agar	1.6%	12
Indicator strain (OD_600_ 10)	N/A	0.375
Sterile ddH_2_O	N/A	2.125
**Total**	N/A	**30 mL**

***Note:*** For optimal results, it is important that the 4% agar solution is heated thoroughly before use to ensure that no clumps or inconsistencies are present.

***Note:*** For optimal results, it is important to limit air bubbles in the plates. 1) After autoclaving or heating the 4% agar in the microwave, leave it on the bench to cool down. This allows air bubbles to go out of the solution. Gently swirl it a few times. 2) Nutation instead of vortexing the media mixture in the falcon tube reduces the introduction of air bubbles in your agar media, which will increase the quality of your halo assay images.

***Note:*** The optimal amount of sensitive strain added may vary depending on the strains used. The amount displayed in the table is used for *S. cerevisiae* BY4741.

***Note:*** Do not wait too long with the next steps to limit growth differences between the embedded strain and the killer strain.

***Optional:*** If needed, methylene blue can be added to the plates to increase the contrast of the zones of inhibition (final concentration 0.001% w/v).

4. Day 2: Spot the strains of interest on top of the assay plates.
  a. From each strain of interest, spot 5 μL of culture (OD_600_ of 10) on top of the assay plate. Take into account appropriate distances between the spots such that any created zones of inhibition will not overlap.
  b. Let the spots dry, for example by opening the plate next to the flame.
  c. Incubate the assay plate at the appropriate conditions for toxin activity (e.g., 25°C).
5. Day 4-5: Data analysis.
  a. Take images of the formed zones of inhibition using an available setup in your laboratory.
  b. ImageJ can be used to quantify the sizes of the zones of inhibition.

***Optional:*** Instead of spotting 5 μL of cell cultures, one can test killer activity of larger volumes of liquids by creating a well in the agar using a sterile metal cylinder. After removal of the cylinder, a volume of spent culture medium or toxin concentrate can be pipetted into the well. Alternatively, filter paper discs have also been used as a means to apply testing solutions (as described in:^5^).

***Optional:*** If many killer strains need to be tested, they can be arrayed into microtiter plates and pinned onto an assay plate containing a sensitive indicator strain using a manual or robotic replicate pinner.^6^

### Part 2: Assessing immunity and resistance phenotypes in liquid culture using a microtiter plate growth assay

**Timing: 4 days**

**Figure 4:**
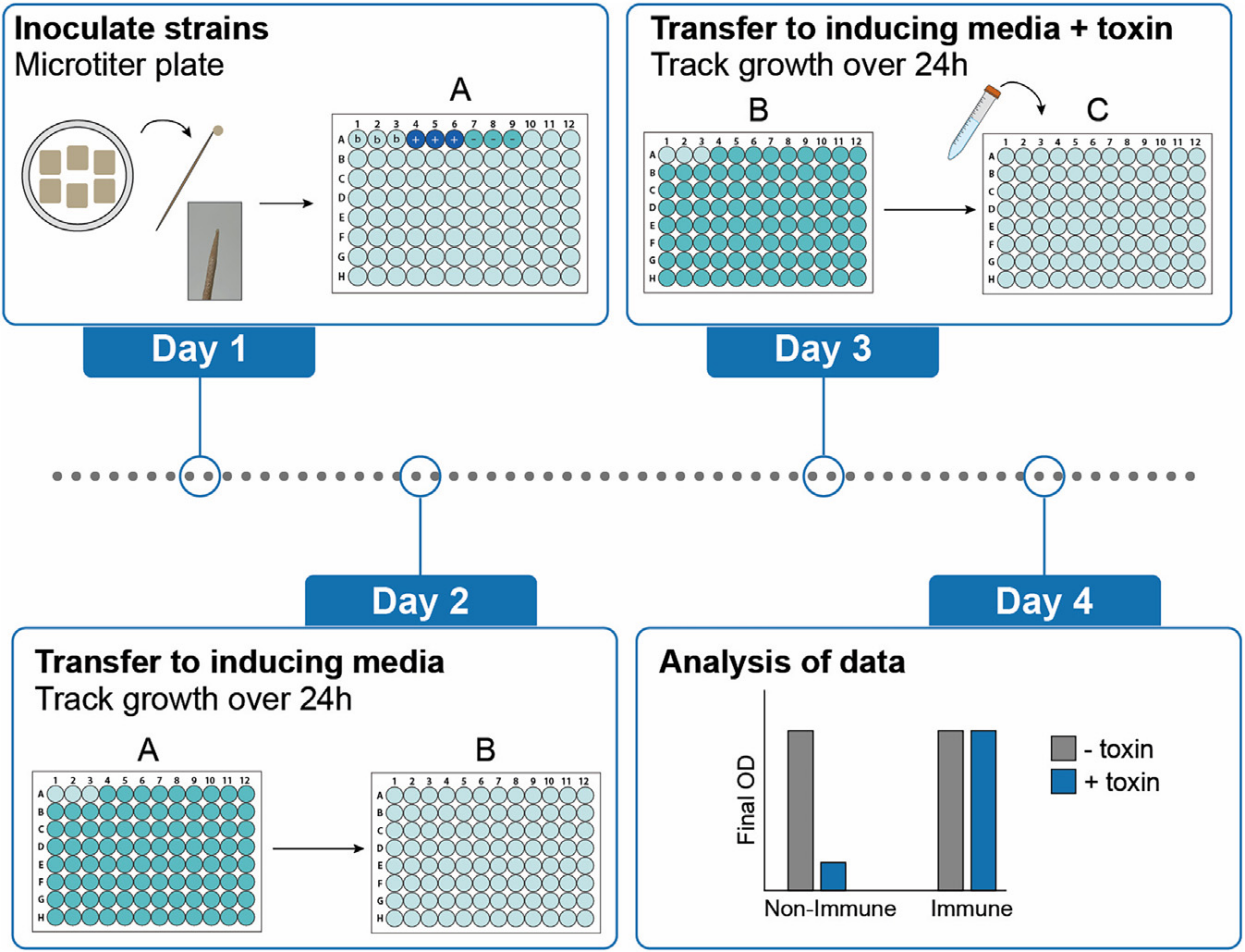
Process of performing a microtiter plate growth assay Day 1: Inoculate small, similar amounts of the patched yeast strains into the wells of a microtiter plate (‘A’). Include blank wells (b), and positive (+) and negative (−) controls. Incubate the plate overnight. Day 2: From the source plate (‘A’), transfer culture to a fresh plate containing inducing media and record the growth for 24 h as a reference. Day 3: From plate B, transfer culture to plate C, which contains medium supplemented with killer toxin. Record the OD_600_ for 24 h. Day 4: Analysis of data indicates growth differences between non-immune and immune strains in the presence or absence of toxin.

While the halo assay determines active toxin secretion and sensitivity phenotypes (of an indicator yeast) on solid media, this section describes an assay for determining sensitivity phenotypes in liquid media using externally supplied toxin (Figure 4). At this stage, the prepared killer toxin concentrate is used (see before you begin). Here, we assume that a potential immunity factor is expressed using a galactose-inducible promoter.

***Note:*** Strains are grown in the presence or absence of the toxin and their growth is recorded by measuring OD_600_ over 24 h. Strains that grow in the presence of the toxin are considered immune, while those that do not grow are considered to be sensitive.

***Note:*** Prior to the assay, a dilution series of the 100X concentrated toxin is tested against a sensitive strain to establish the minimal inhibitory concentration (MIC) and determine the final toxin concentration for use in assays (see Figure 6B, left panel); in our case, the MIC was approximately 2X of killer toxin concentrate, but the final concentration of toxin in the assay was set to 10X to ensure a robust growth difference between sensitive and immune strains, and to account for batch-to-batch variation in killer toxin concentrate.

6. Day 1: Create a source plate ‘A’ containing the strains of interest, including positive and negative controls.
  a. Prepare sufficient 1X buffered SD_suc_ media to fill the appropriate number of wells of a 96-well microtiter plate (200 μL per well). Include 3 ‘blank’ wells where just media without the cells is added.
  b. Using sterile toothpicks, inoculate biomass from the patches into the wells, with the desired number of replicates for each strain.
  c. Cover the plate (plate A) with a breathable membrane and incubate overnight (25°C) in a standing incubator.

***Note:*** This step allows the culture in each well to reach a similar saturated OD_600_ overnight and increases the reproducibility of the results.

**CRITICAL:** For optimal results, it is important to inoculate small, similar amounts of biomass per well to limit large differences in cell number per well (see Figure 4**- Day 1**).

7. Day 2: Transfer the cultures to fresh inducing medium and record the OD_600_ over 24 h.
  a. Prepare 1.05X buffered SD_suc_ medium supplemented with 1% galactose.
  b. In new wells, add 190 μL of this medium (plate ‘B’).
  c. Using a multichannel pipette, resuspend the wells of plate A and transfer 10 μL of cultures from plate A to plate B.
  d. Cover plate B with a breathable membrane and record the OD_600_ in a plate reader for 24 h, at 25°C, under continuous shaking.

***Note:*** This step records growth curves in inducing media, indicating any effects that protein expression (i.e. expression of an immunity factor that is tested) may have on cell fitness in absence of the toxic compound that will be added in the next step. In case expression of immunity factors is tested, this incubation simultaneously allows time for expression of the immunity factor and establishing immunity before exposure to the toxin.

8. Day 3: Transfer the cultures to fresh inducing medium supplemented with toxin and record the OD_600_ for 24 h.
  a. Prepare 1.05X buffered SD_suc_ medium, supplemented with 1% galactose and 10X of the killer toxin concentrate.
  b. In new wells, add 190 μL of this medium (plate ‘C’).
  c. Using a multichannel pipette, resuspend and transfer 10 μL of cultures from plate B to plate C.
  d. Cover plate C with a breathable membrane and record the OD_600_ in a plate reader for 24 h, at 25°C, under continuous shaking.

***Note:*** In this step, the effect of the toxin on the growth of the strains is recorded over 24 h.

***Note:*** Note that the amount of killer toxin concentrate to add can differ depending on the killer yeast strain and killer toxin used for preparing the killer toxin concentrate. This can be determined by using a dilution range of the toxin in step 8a, defining at what concentration there is a robust difference in cell growth between a resistant and a sensitive strain (see Figure 6B).

9. Day 4: Data analysis.
  a. Subtract the blank values (of media only).
  b. Since the later OD_600_ values are typically outside the linear range of the photodetector, all OD_600_ values need to be corrected to account for the saturation of the sensor, as previously described.^7^
  c. Plot the graphs using an appropriate program (e.g., Excel, Prism) - See expected results section.

***Note:*** If no microtiter plate reader is available, the cultures can in principle also be grown in culture tubes or shake flasks instead of microtiter plates, scaling the volumes proportionally. In this case, take a sample at the end time point to determine the final OD_600_, or prepare sufficient culture volumes such that multiple samples can be taken over several time points to create a growth curve.

**Figure 5:**
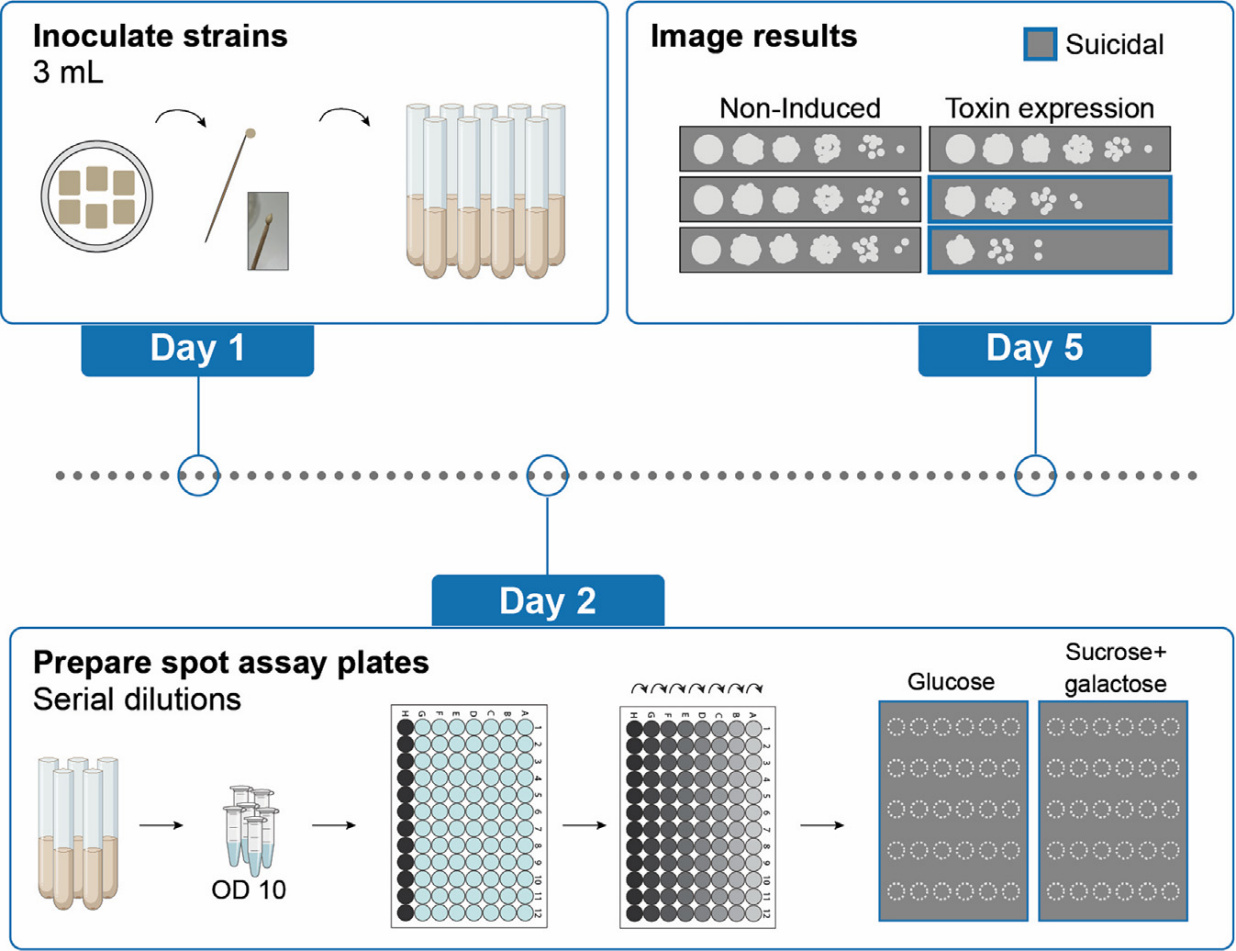
Process of performing a spot assay Day 1: Inoculate the strains of interest in non-inducing medium. Day 2: After normalizing the OD_600_ to 10, prepare a 10-fold serial dilution of the overnight cultures. Spot each dilution onto repressing (SD_gluc_) and inducing (SD_suc_ + galactose) agar plates. Day 5: After incubation of the plates, suicidal strains are recognized by reduced growth on the induced plate compared to a control strain.

**Figure 6:**
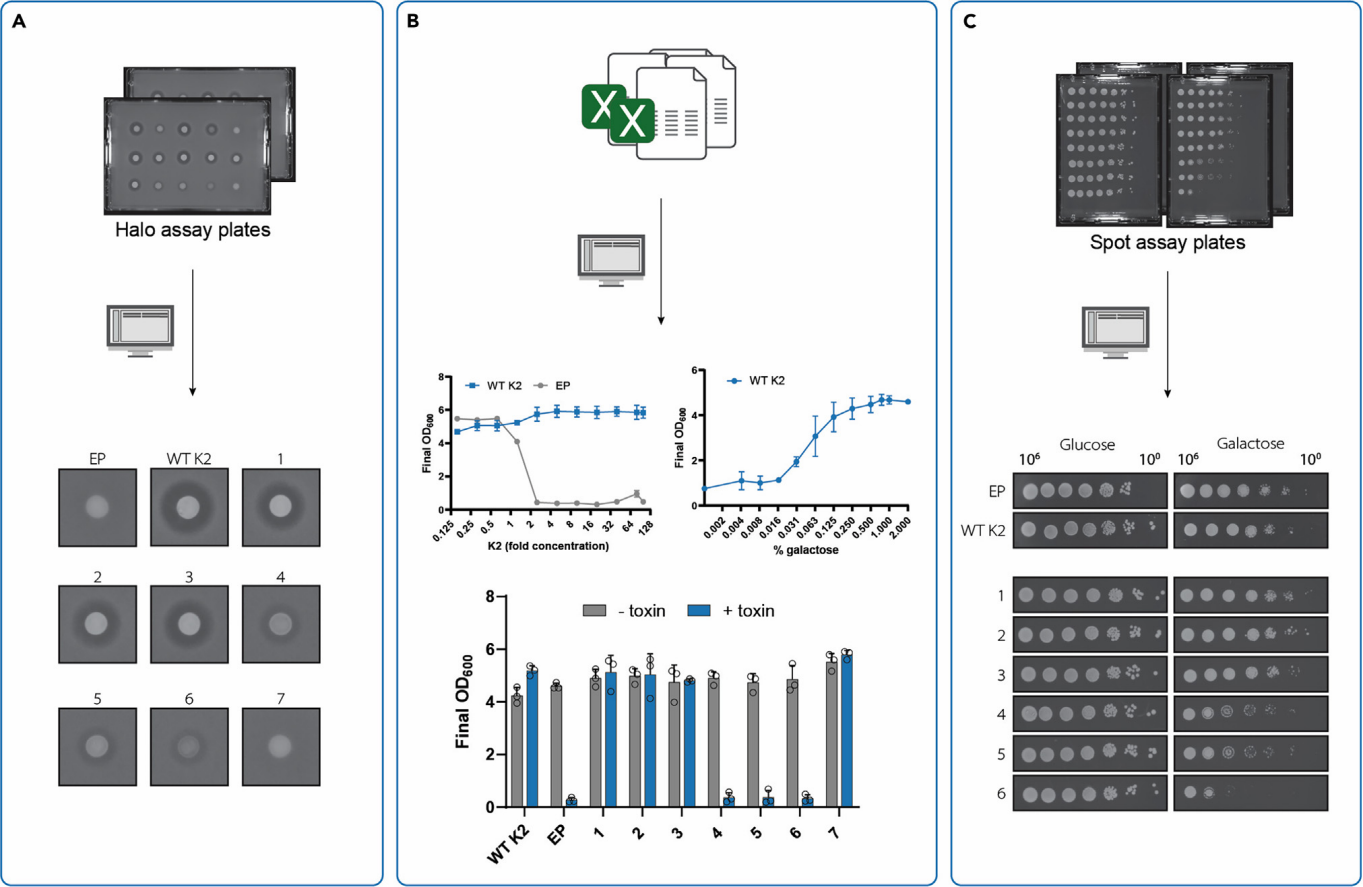
Expected outcomes for the assays (A) Halo assay in SD_suc_ medium containing 1% galactose (image is representative of a duplicate experiment). (B) Microtiter plate liquid culture assay. Left panel: WT K2 and EP strains were grown in SD_suc_ medium containing 1% galactose and varying concentrations of killer toxin concentrate. Right panel: WT K2 was grown in SD_suc_ medium containing 10X of killer toxin concentrate and varying concentrations of galactose. Bottom panel: Resulting final OD_600_ values of growth of the indicated strains in SD_suc_ medium containing 1% galactose with or without 10X of killer toxin concentrate. The graphs show the mean final OD_600, with error bars indicating_ ± 1 standard deviation. (C) Spot assay of a 10-fold culture dilution series (∼10^6^ cells to 10^0^ cells) on SD_gluc_ medium and SD_suc_ with 1% galactose (image is representative of a duplicate experiment). EP: empty plasmid control. WT K2; wild-type K2 toxin under the pGal1 promoter. The other strains express K2 toxin variants 1-7 under the pGal1 promoter. Parts of this figure were adapted from^1^ which is published under a CC-BY license permitting the reproduction of figure parts if cited.

### Part 3: Assessing suicidal phenotypes by spot assay (serial dilution assay)

**Timing: 2 days + 3 days incubation**

Where the halo assay determines toxicity phenotypes, and the microtiter plate assay assesses immune phenotypes, this part of the protocol addresses the interplay between toxicity and self-protection by describing how to assess potential suicidal phenotypes in killer yeast using a spot assay (Figure 5). Here, we also make use of a galactose-inducible promoter.

***Note:*** When a yeast killer toxin is expressed without functional self-protection, it leads to loss of fitness. To perform the assay, starting cultures are cultivated in non-inducing, buffered medium (SD_suc_). A serial dilution of these cultures is generated and spotted onto either repressing (SD_gluc_) or inducing (SD_suc_ + galactose) plates. The growth patterns observed between the induced plates, compared to the non-induced plate, reveal potential fitness differences between the yeast strains.

10. Day 1: Inoculate the strains of interest, including positive and negative controls.
  a. Prepare non-inducing 1X buffered SD_suc_ media (for media recipe see: Materials and Equipment **Setup**)
  b. Prepare culture tubes containing 3 mL of this medium for the appropriate number of strains and replicates.
  c. Inoculate the strains from the respective patches using sterile toothpicks.
  d. Incubate the cultures overnight at 25°C, 200 rpm.
11. Day 2: Adjust the OD_600_ of all cultures.
  a. Determine the OD_600_ of all cultures using a spectrophotometer.
  b. Adjust the cell concentration of all overnight cultures to an OD_600_ of 10.
    i. Transfer 1 mL of the cultures into Eppendorf tubes.
    ii. Pellet the cells (i.e., 5000 *g* for 2 min).
    iii. Adjust the OD_600_ by removing an appropriate volume of the spent culture media (or adding an appropriate volume if the OD_600_ is above 10).
    iv. Vortex the tubes to resuspend the cell pellet.

***Note:*** For consistent results, it is important that the overnight OD_600_ values of the strains are similar. If the promoter is leaky, strongly suicidal strains may already show a decrease in OD_600_.

12. Day 2: Prepare assay plates (non-inducing and inducing).
  a. Prepare 30 mL solid media for each plate, one set of plates containing glucose as the carbon source (SD_gluc_), and another set of plates containing buffered media with sucrose as the carbon source (SD_suc_) and supplemented with 1% galactose (for the media recipe see: materials and equipment Setup).
  b. Pour the plates and let them solidify on the bench.

**Figure 7:**
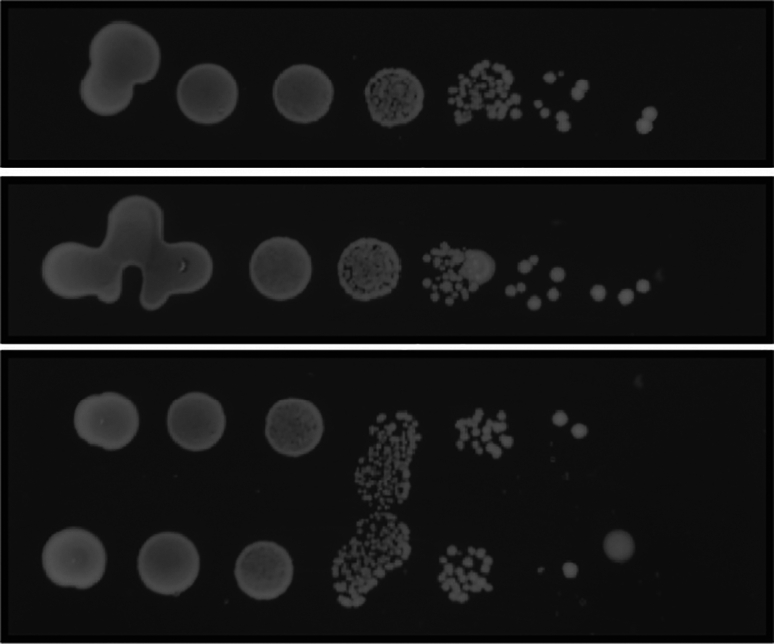
Dispersed and merged spots in a serial dilution assay due to condensation droplets

**CRITICAL:** For optimal images, do not stack the plates to prevent formation of condensation droplets on the agar surface. It may help to limit the time between step 12 and step 14. In our experience, these droplets, or the marks they leave after drying, lead to dispersion of the culture droplet at those locations, leading to non-circular shapes or risk of neighboring culture droplets fusing together (see Figure 7).

13. Day 2: Prepare a 10-fold serial dilution for each strain.
  a. Take a 96-well microtiter plate and rotate it 90°.
  b. In the first column, add approximately 150 μL of the culture (OD_600_ = 10).
  c. In the other columns, add 180 μL of sterile ddH_2_O.
  d. Using a multichannel pipette, transfer 20 μL from the first column into the second, pipette up and down several times to mix, transfer 20 μL to the next column, etc.

**CRITICAL:** After preparing the serial dilution, yeast cells will start to settle on the bottom of the wells. Therefore, swiftly continue with step 14, or ensure the cultures are properly resuspended by pipetting the well up and down before transferring culture to the assay plate.

14. Day 2: Spot the serial dilution onto the assay plates.
  a. For each strain, spot 5 μL of each well next to each other onto both the inducing and non-inducing plate.
    ***Note:*** While a multichannel pipette could speed up this process, in our experience this can lead to ‘splashing’ and therefore additional colony spots on the plates. To prevent this, cultures should be spotted individually with a regular pipette.
  b. Let the spots dry, for example by opening the plate close to a flame.
  c. Incubate the plates at the appropriate conditions (e.g., 25°C for K2 toxin activity).
    ***Note:*** If we assume around ∼2∗10^7^ cells in each OD_600_ unit per mL of culture, then a 5 μL spot of an OD_600_ of 10 will result in ∼1∗10^6^ spotted cells. This is an estimation for our strain and the number of cells per OD_600_ unit varies between strains.
    ***Note:*** Use appropriate distance between spot series of different strains, such that the potential toxin secretion of one will not affect the growth of another.
15. Day 5: Take images of the plates.

## Expected outcomes

This protocol is designed to provide a comprehensive approach to study killer yeast phenotypes using three complementary assays. Together, these can determine toxic, immune, and suicidal phenotypes of a killer yeast. We used this protocol for studying the K2 killer toxin in *S. cerevisiae*.^1^ Typical results are exemplified by wild-type K2 toxin and K2 variants 1-7, as shown in Figure 6, all expressed using a galactose-inducible promoter. The wild-type K2 precursor encodes both the K2 toxin as well as a self-protective immunity factor.

All strains except for those harboring an empty plasmid (EP) and those expressing variant 7 create a zone of inhibition on plates containing an embedded sensitive *S. cerevisiae* strain (Figure 6A). By growing either the strain expressing wild-type K2 (WT K2) or EP in the presence of varying concentrations of the K2 toxin concentrate, we determined that toxin concentrations above 2X elicit a clear difference in sensitivity of both strains (Figure 6B, left panel). Growing WT K2 in the presence of 10X toxin while varying the galactose concentration indicates that with 1% galactose, the strain produces sufficient immunity factor molecules to grow (Figure 6B, right panel). Strains expressing variants 4, 5 and 6 cannot grow in media containing 10X toxin and 1% galactose, and thus do not contain a functional immunity factor, whereas the strain expressing variant 7, which did not produce active toxin, does produce a functional immunity factor and can grow (Figure 6B, lower panel). The fact that the strains expressing variants 4, 5 and 6 secrete active toxin (Figure 6A), but do not contain a functional immunity factor against this toxin (Figure 6B, bottom panel), implies that these strains may be suicidal. This suicidal phenotype is confirmed in the spot assay, where these variants show a growth reduction on the plate containing 1% of the inducer galactose (Figure 6C).

a. Expected outcomes halo assay: Clear zones of inhibition (halo) are formed around the spotted killer strains, indicating secretion of active toxic compounds that inhibit growth of the strain embedded in the agar. The halo indicates that the embedded strain is sensitive to the toxin produced by the spotted strain. The size of the zone of inhibition will depend on the toxin used and the sensitivity of the used indicator strain. Strains that do not produce toxins to which the embedded strain is sensitive, do not show formation of a zone of inhibition around the spot. When testing killer toxin variants, the size of the halo relates to the remaining toxin functionality. This functionality is the result of various factors, including the efficiency in translation, production and secretion of the toxin, as well as toxin stability, diffusion through the medium and its interaction with target cells.

b. Expected outcomes microtiter plate growth assay: To test if a strain is sensitive to a toxin or immune, besides the halo assay, a liquid culture assay can also be used in which the strain is cultivated in the presence or absence of the toxin. Strains that are sensitive to the toxin will show inhibited growth in the presence of toxin, compared to the normal growth in its absence. Strains that are completely resistant or immune will grow normally in both conditions. Varying the galactose concentration or toxin dose, MIC and dose-response analysis is possible. It is best to define the growth phenotypes for resistance and sensitivity using appropriate positive and negative controls (see Figure 6B, left and right panel), before designing the assay conditions. The MIC, inducer concentration (if used), the toxin, strains, and immunity factor under study may require altered parameters. Besides complete resistance and immune phenotypes, a range of partially immune phenotypes can also be observed (Figure 6B, right panel). The assay can distinguish more subtle differences in sensitivity than the halo assay.

c. Expected outcomes spot assay: Presence or absence of self-protection can be assessed in a spot assay using serial dilution of a culture. On repressing plates (SD_gluc_), normal growth is expected since toxin expression is not induced. If all tested strains show the same growth, this is an indication that the dilution series contained equal cell numbers and that the effects observed on the induced plate (SD_suc_ + galactose) can be compared to each other. If growth on the inducing plates is reduced or absent, this is an indication that the yeast lacks self-protection against the toxin it produces. The EP and WT K2 are both not suicidal, because they either do not produce toxin (EP) or produce toxin together with a functional self-protection factor (WT K2), and show growth in the spots of ∼10^0^ and/or ∼10^1^ cells, with dense growth in spots with a higher number of cells. Reduced growth was defined by those strains that only show higher density in the first spots (∼10^6^ to 10^4^) which greatly reduced growth in the spots with a higher dilution (10^3^-10^0^), indicating that a substantial amount of the cells is unable to grow – here due to self-killing.

## Limitations

Several of the parameters may require optimization when applied to other toxins and killer yeast or sensitive indicator strains, such as the used number of cells in each assay, physicochemical parameters required for toxin activity (pH and temperature), and incubation length and temperatures. However, the presented protocol provides a robust scaffold for analyzing toxic, immune and suicidal phenotypes in yeast. For replicable results, key factors such as the initial cell numbers and growth phase of the cultures need to be carefully controlled. The **halo assay** is particularly sensitive to differences in growth rate between the embedded sensitive indicator and spotted killer strains. If the embedded strain grows considerably faster, it may quickly overgrow and a clear killing phenotype may not become visible. A microtiter plate assay may in such cases be an alternative option, where a killer toxin concentrate is prepared and added to a culture of the sensitive indicator strain as outlined in Part 2. A successful halo assay depends on the choice of an appropriate sensitive strain, and knowledge on other toxin activity requirements such as the optimal pH and temperature. Within the **microtiter plate assay**, the inoculated cell number is a critical factor that can influence the sensitivity of the assay. Since toxin is added only at the start of the incubation, it needs to be sufficiently stable in the culture medium for the incubation length. For the **spot assay**, it may not be possible to observe small fitness differences between strains. The assay requires an inducible (e.g., galactose) or repressible (e.g., pH, temperature, glucose) phenotype such that differences due to active toxin production can be determined. Since nutrient composition of the media may also influence fitness of the strain independent of toxin expression, it is important to incorporate appropriate controls.

## Troubleshooting

### Problem 1

No halo is formed around the spotted strains in the halo assay.

### Potential solution

Check whether the embedded strain is growing properly within the agar, and the spotted strain on top of the agar. If the 4% agar was too hot it can cause lack of growth of the embedded strain, resulting in a transparent plate. Verify that the correct strains and media conditions are used (pH, temperature) and that the sensitive strain is indeed sensitive to the specific toxin and that a toxin is secreted. The latter can be verified by preparing a toxin concentrate from the spent supernatant of the killer yeast and exposing the sensitive strain to it in a microtiter plate growth assay as outlined in Part 2 of this protocol.

### Problem 2

The embedded strain is too dense (halo barely visible) or not dense enough (little contrast in the halo).

### Potential solution

Optimize the concentration of seeded sensitive cells. The optimal number of cells depends on the growth rate of the strain compared to the spotted strain, and the optical density to which the strain grows in the used medium composition. The incubation time or temperature can also be optimized.

### Problem 3

I want to perform a halo assay with a liquid solution containing toxin instead of spotted cultures of toxin producers.

### Potential solution

This is possible by using metal cylinders that can be sterilized in ethanol and by flaming. They can be placed on top of a thin layer of solid media, and the solid media containing the sensitive strain can be poured around it. Removing the cylinders after the plate has solidified yields holes that can be filled with the liquid. If the liquid contains a toxin to which the embedded strain is sensitive, this will create a zone of inhibition around the hole.

### Problem 4

Large error bars in the microtiter plate reader assay.

### Potential solution

It is important to inoculate similar amounts of biomass at the first step (and not a too large volume). Ensure that cells have not settled to the bottom of the 96-wells plate throughout the process and fully resuspend the cells by pipetting up and down before transfer from plate to plate.

### Problem 5

No inhibition observed in the microtiter plate reader assay.

### Potential solution

Confirm that the concentrated toxin is active by re-testing it against a known sensitive strain – this can also be done in a halo assay. Check that the correct medium conditions and final toxin concentration are used. Ensure that the toxin concentrate is stored in such a way that its activity is maintained.

### Problem 6

Uneven growth across spots and cross-contamination between neighboring spots in the spot assay.

### Potential solution

Make sure to use appropriate spacing between the spots. Limit the number of air bubbles and other irregularities in the agar surface. It is best to prepare agar plates fresh. This prevents the formation of condensation droplets on the plates that could otherwise lead to dispersion of the spotted culture droplets (see Figure 7). Allow the spots to dry before turning the plates upside down for plate incubation.

## Resource availability

### Lead contact

Further information and requests for resources and reagents should be directed to and will be fulfilled by the lead contact, Sonja Billerbeck (s.k.billerbeck@rug.nl).

### Technical contact

Technical questions on executing this protocol should be directed to and will be answered by the technical contact, Sonja Billerbeck (s.k.billerbeck@rug.nl).

### Materials availability

This study did not generate new unique reagents.

### Data and code availability

The data that support this protocol are available from the corresponding author, SB, upon reasonable request.

## Acknowledgments

The work was funded by an FSE grant from the University of Groningen (SB) and by grants OCENW.XS3.069 (S.B.), OCENW.M20.250 (S.B.), and OCENW.XS21.2.060 (S.B.) from the Dutch Research Council (NWO).

## Author contributions

R.C.P. and S.B. developed the protocol, R.C.P. wrote the manuscript, and S.B. edited the manuscript.

## Declaration of interests

The authors declare no competing interests.
